# Surveillance and control of SARS‐CoV‐2 in mustelids: An evolutionary perspective

**DOI:** 10.1111/eva.13310

**Published:** 2021-11-06

**Authors:** Adriana V. Díaz, Martin Walker, Joanne P. Webster

**Affiliations:** ^1^ Department of Pathobiology and Population Sciences Royal Veterinary College University of London Herts UK

**Keywords:** biotic hub, evolution of virulence, farmed mink, one health, reverse zoonoses, SARS‐CoV‐2

## Abstract

The relevance of mustelids in SARS‐CoV‐2 transmission has become increasingly evident. Alongside experimental demonstration of airborne transmission among ferrets, the major animal model for human respiratory diseases, transmission of SARS‐CoV‐2 within‐ and/or between‐commercial mink farms has occurred and continues to occur. The number of mink reared for the luxury fur trade is approximately 60.5 million, across 36 mustelid‐farming countries. By July 2021, SARS‐CoV‐2 outbreaks have been reported in 12 of these countries, at 412 European and 20 North American mink farms. Reverse zoonotic transmission events (from humans to mink) have introduced the virus to farms with subsequent extensive mink‐to‐mink transmission as well as further zoonotic (mink‐to‐human) transmission events generating cases among both farm workers and the broader community. Overcrowded housing conditions inherent within intensive mink farms, often combined with poor sanitation and welfare, both guarantee spread of SARS‐CoV‐2 and facilitate opportunities for viral variants, thereby effectively representing biotic hubs for viral transmission and evolution of virulence. Adequate preventative, surveillance and control measures within the mink industry are imperative both for the control of the current global pandemic and to mitigate against future outbreaks.

## 1 INTRODUCTION

Pathogens are capable of rapid evolution in response to human activities (Ewald, 1998). No other human activity has had a more remarkable effect in shaping civilization than agriculture (Ehrlich & Ehrlich, 2013): it has sustained increasingly large human populations, altered a range of ecosystems and set the conditions for otherwise unlikely interspecies encounters. Both animal farming and animal trade lead to the aggregation of host species that can maintain and enhance emergence of human‐shared pathogens and serve as a bridge between wildlife and humans. All three highly infectious human coronaviruses (hCoVs) that have emerged in the 21^st^ century are thought to have jumped to human hosts after sequential spillover from their likely bat reservoir to domesticated or marketed mammals.

The first emergent hCoV of this century, severe acute respiratory syndrome coronavirus (SARS‐CoV or SARS‐CoV‐1), recombined in masked palm civets—which are caught in the wild and/or farmed and sold in markets as exotic food (Shi & Hu, 2008)—before infecting humans during two independent animal‐to‐human transmission events in South China in 2002–2003 and 2003–2004 (Kan et al., 2005; Shi & Hu, 2008). The second emergent hCoV, Middle East respiratory syndrome coronavirus (MERS‐CoV), a highly prevalent virus of dromedary camels, spilled over to humans in Saudi Arabia in 2012 (Cui et al., 2019), and thousands of human cases of a particular MERS‐CoV lineage have since been linked to direct or indirect contact with camels (Chafekar & Fielding, 2018). The third emergent hCoV, severe acute respiratory syndrome coronavirus 2 (SARS‐CoV‐2), was identified in a cluster of atypical pneumonia cases connected to a live animal market in Wuhan, Central China, at the end of 2019 (Chen et al., 2020; Lu et al., 2020).

The hypothesized adaptation of SARS‐CoV‐2 from bats to an intermediary host or hosts (Andersen et al., 2020; Oude Munnink et al., 2021), and from this unclarified link to humans (Latinne et al., 2020; WHO Team, 2021), has resulted in sustained high levels of human‐to‐human transmission and a global pandemic of coronavirus disease (COVID‐19). The rapid expansion of SARS‐CoV‐2 among human populations on every continent (Williams & Burgers, 2021) has, in turn, created opportunities for the virus to adapt to new hosts, particularly domestic and farmed animals. Reverse zoonotic transmission from humans to pet dogs (*Canis lupus familiaris*) and cats (*Felis catus*), and recently, a pet ferret (*Mustela putorius furo*) has been reported (OIE, 2021b), although experimental evidence of high susceptibility in companion animals to date only implicates cats, golden Syrian hamsters (*Mesocricetus auratus*) and ferrets, with documented additional intra‐species transmission in the latter three (OIE, 2021a). Susceptibility to SARS‐CoV‐2 has been discounted in cattle, chicken, ducks, turkeys and pigs (OIE, 2021a), but other farmed animals such as rabbits (*Oryctolagus cuniculus*) and raccoon dogs (*Nyctereutes procyonoides*) have proved moderately and highly susceptible to experimental infection, respectively, with evidence of subsequent transmission between racoon dogs (OIE, 2021a). Most notably, however, two members of the mustelid family, ferrets and American mink (*Neovison vison*, see Box 1), are not only highly susceptible to experimental infection but can also acquire and transmit the virus naturally (OIE, 2021a).

BOX 1 **Facts about American mink**

**FIGURE 1:**
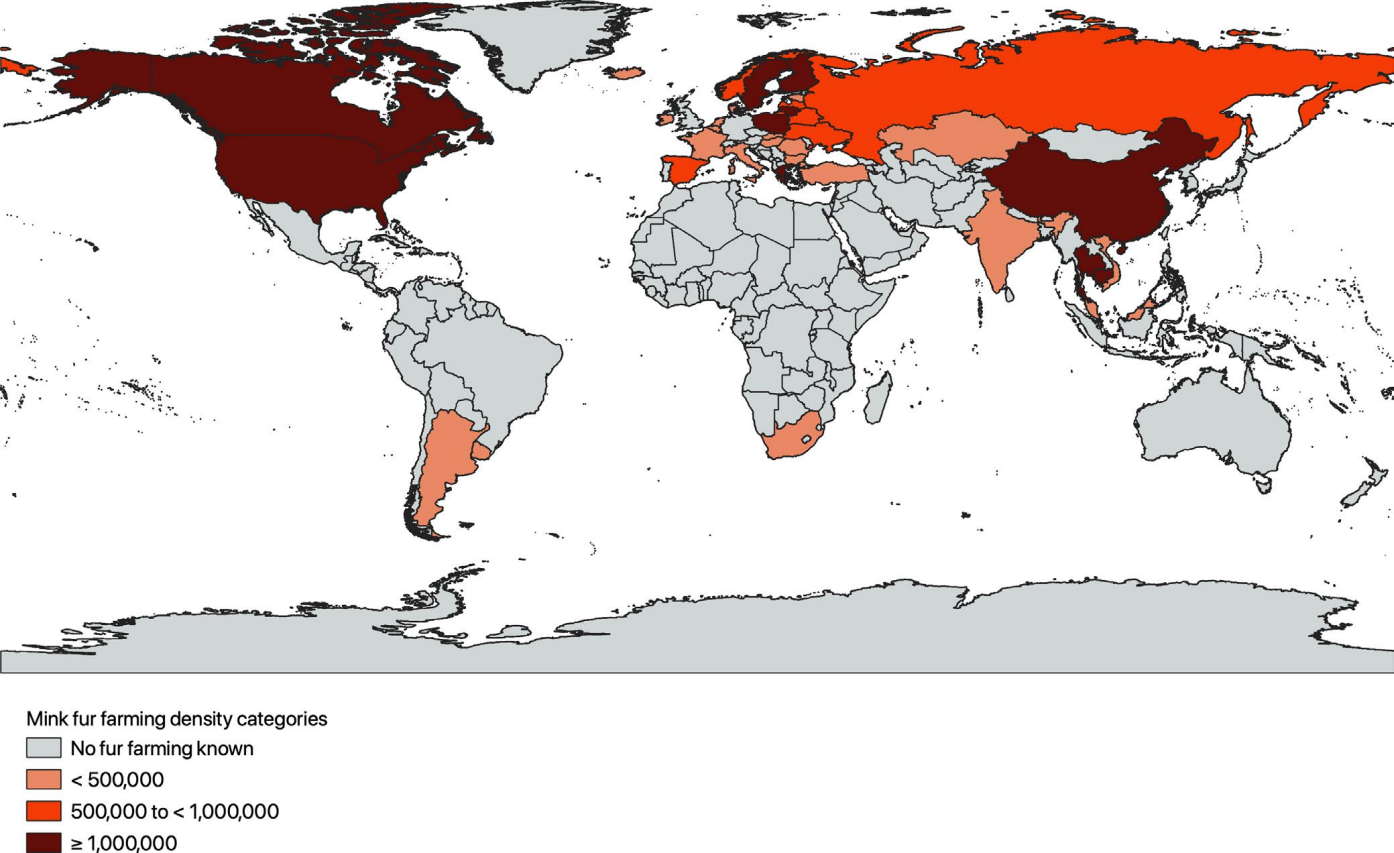
Farmed mink density worldwide as of 20 January 2021

- *Neovison vison*, also known as *Mustela vison*, are fur‐bearing, semi‐aquatic mammals which are part of the largest family in the Carnivora order, Mustelidae, that includes weasels, otters, badgers, wolverines, martens and ferrets.
- Native to the United States and Canada in North America, American mink are now found in other continents including South America, Europe and Asia (see Figure 1).
- These solitary and territorial animals are opportunist predators of rodents, waterbirds, crustaceans, amphibians, reptiles and fish. With such a generalist carnivore diet, they adapt quickly to a range of aquatic and riparian habitats, making them a very successful invasive species (Palazón & CABI, 2014).
- Territorial encroachment of this invasive species impacts native wildlife. For example, decimation of water vole (*Arvicola amphibius*) populations in the UK and European mink (*Mustela lutreola*) in Eurasia is not least due to American mink predation and competition, respectively (Martin & Lea, 2020).

Overview of the global density of farmed mink using information from the SARS‐CoV‐2 risk assessment on fur farms gathered by the Joint FAO–OIE–WHO Global Early Warning System for health threats and emerging risks at the human–animal–ecosystems interface (WHO et al., 2021). SARS‐CoV‐2 susceptible animals are commercially farmed for fur in 36 countries in the world including the following: Argentina, Belarus, Belgium, Bulgaria, Cambodia, Canada, China (People's Rep. of), Denmark, Estonia, Finland, France, Greece, Hungary, Iceland, India, Ireland, Italy, Kazakhstan, Latvia, Lithuania, Malaysia, Netherlands, Norway, Poland, Romania, Russian Federation, Slovakia, South Africa, Spain, Sweden, Thailand, Turkey, Ukraine, United States of America, Uruguay and Vietnam.

Map created using the Free and Open Source QGIS 3.16.6‐Hannover.

## 2 SARS‐CoV‐2 IN MUSTELIDS

Ferrets are one of the major established animal models for human respiratory diseases (Muñoz‐Fontela et al., 2020), used to evaluate the airborne transmission potential of influenza viruses (ECDC, 2020), being natural hosts for both type A and B human influenza (Maher & DeStefano, 2004). Moreover, they are used to investigate the pathogenesis and transmission of human respiratory syncytial virus (hRSV) and SARS‐CoV‐1 (Stout et al., 2021). Accordingly, ferrets are also key animal model hosts for experimental studies on SARS‐CoV‐2 pathogenicity and transmission (ECDC, 2020; Kim et al., 2020; Muñoz‐Fontela et al., 2020; Stout et al., 2021), presenting mild clinical signs of upper respiratory tract infection including fever and nasal discharge (Stout et al., 2021). In addition to efficient SARS‐CoV‐2 infection via ferret‐to‐ferret direct contact (one to three days after exposure), indirect airborne transmission between animals housed in cages 10 cm apart (three to seven days postexposure) has been documented experimentally (Richard et al., 2020).

Transmission of SARS‐CoV‐2 among American mink (*Neovison vison*), farmed for its fur (see Figure 1 and Box 2), is both highly efficient and prevalent, with ongoing outbreaks reported (up to 6 July 2021) in commercial units from 12 countries, in order of first occurrence: the Netherlands, Denmark, Spain, USA, Sweden, Italy, Greece, France, Lithuania, Canada, Poland and Latvia (OIE, 2021b). Further outbreaks are likely to have been missed amid high background morbidity and mortality levels inherent to such intensive production system (Compo et al., 2017; Honoré et al., 2020; Wang et al., 2014; Wilson et al., 2015). Mink infected with SARS‐CoV‐2 display a range of clinical signs from asymptomatic infection, watery to mucoid nasal discharge and/or inappetence, to severe dyspnoea and sudden deaths (Molenaar et al., 2020), with varying morbidity and mortality among farms (Boklund et al., 2021; Hammer et al., 2021; OIE, 2021a).

BOX 2 **The mink industry**

- Only American mink (*Neovison vison*) are farmed, and not the smaller European mink (*Mustela lutreola*), a distant critically endangered cousin.
- These small mustelids have been selectively bred for their attractive fur since 1866 (Fur Commission USA, 2014).
- Mink hides continue to be used as luxury clothing items: 15–20 pelts can make a short coat whereas 20–30 animals can make a long coat (EIA, 2020).
- Mink regularly escape from farms and have established long‐standing feral colonies in the wild (ECDC, 2020; Palazón & CABI, 2014).
- Reported behavioural changes such as fearfulness, self‐mutilation and infanticide reflect welfare shortcomings (Xia et al., 2020).
- The environmental impact of producing 1 kg of mink fur has a five times higher carbon footprint than that involved in producing 1 kg of wool (Xia et al., 2020).
- The production cycle of mink farms involves a closed system, meaning all steps of production from birth to slaughter (breeding, whelping, weaning, growing), and pelting (skinning), take place within the same farm, over 1 year (ECDC, 2020).
- Between 22 and 23 countries in Europe harvest half of the world's mink fur: over 27 million pelts per year are produced by 2750 European mink farms according to some estimates (ECDC, 2020; Koopmans, 2021), or around 30 million pelts produced in 5000 European farms according to other estimates (WHO et al., 2021).
- In 2018, the biggest fur producers in Europe were Denmark (17.6 million animals), followed by Poland (5 million), the Netherlands (4.5 million), Finland (1.85 million), Greece and Lithuania (both 1.2 million) (WHO et al., 2021). In the former four fur‐producing countries, there were approximately 1200, 300, 125 and 900 mink farms, respectively (Fenollar et al., 2021).
- Other significant mink producers in the world include China (20.7 million animals in 2018), the United States of America (3.1 million in 2018) and Canada (1.7 million in 2018) (WHO et al., 2021). The number of mink farms in these countries was estimated to be around 8000 and 245, respectively, in China and the USA (Fenollar et al., 2021), and more than 200 in Canada in 2017 (CMBA, 2020).
- By 2020, around a third of global mink fur demand was supplied by Denmark (BMJ & Dyer, 2020). China is the biggest mink fur consumer in the world, with a reported annual consumption of 12 million domestic pelts plus a million imported from Denmark, Finland and the United States (EIA, 2020).

Mink farm outbreaks are facilitated by housing conditions which typically consist of adjoining bare wire cages that allow for both free airflow and animal contact within densely populated facilities (ECDC, 2020). The virus, seeded and regularly re‐introduced into farms by staff (Boklund et al., 2021), is thus efficiently maintained and amplified through both mink‐to‐mink contact and likely airborne transmission. Accentuated by the risk of initial misdiagnoses, SARS‐CoV‐2 can spread undetected for weeks within and between mink farms, as was realized in the first two reporting countries in the world, the Netherlands and Denmark (Hammer et al., 2021). In both nations, despite a subsequent integrated response that involved tight biosecurity, targeted culls and active surveillance (see Box 3), transmission chains proved difficult to break and the mode of transmission between some Dutch farms remains elusive (Oude Munnink et al., 2021). The risk of SARS‐CoV‐2 diagnosis among Danish units has been associated with farm size and distance to nearest affected farm, although between‐farm transmission routes beyond direct human contact remain unclear (Boklund et al., 2021). Pertinently, mink‐specific SARS‐CoV‐2 variants evolved in both countries, accumulating mutations such as those in the ORF3a gene (H182Y) and in the spike gene (Y453F) (Hammer et al., 2021). Mutation Y453F (Hodcroft, 2021), along with another three spike protein mutations (an amino acid deletion, H69del/V70del; and two substitutions, I692V and M1129I; SSI, 2020), arose in a particular Danish mink variant known as ‘Cluster 5’, a variant linked to twelve human cases that proved less susceptible to human convalescent neutralizing antibodies, drawing attention to the possible implications of mink‐related SARS‐CoV‐2 mutations on vaccine efficacy and human health (BMJ & Dyer, 2020; Hodcroft, 2021; SSI, 2020; WHO, 2020). Moreover, some of the mink variants potentially increased transmissibility (Hammer et al., 2021) and enhanced mink's role as an amplifying host.

BOX 3 **Varying responses to the farmed mink epidemic: three case studies**

**The Netherlands**

**Initial detection**

The first apparent human‐to‐mink SARS‐CoV‐2 transmission event in the world happened in the Netherlands, a country with an estimated mink herd in excess of 4 million (WHO et al., 2021). Investigations due to respiratory and gastrointestinal signs in two mink farms confirmed SARS‐CoV‐2 infection in mink in late April 2020, with affected farms placed under movement restrictions immediately (Bruschke, 2020a). Further epidemiological examinations concluded the virus had probably circulated for more than a month before detection (Bruschke, 2020b).

**Surveillance and spread**

A surveillance system for the active detection of subclinical and clinical cases in farmed mink was put in place in late May 2020 (Oude Munnink et al., 2021), consisting of mandatory notification, an early warning system linked to mink mortalities, and serological screening of all farms (Bruschke, 2020d). This comprehensive surveillance identified SARS‐CoV‐2 incidence in 13 farms by the beginning of June (Bruschke, 2020d), and a tally of 24 a month later (Bruschke, 2020e). Control measures instated included culls of all animals within infected farms, testing of symptomatic farm staff, intensified biosecurity, personal protective equipment (PPE) for staff exposed to animals and a 10‐day waiting period for employees working in different locations (Bruschke, 2020d). Sequencing analysis implied infected employee‐to‐mink as introduction route for some farms but an unknown route for others, plus viral clustering between several farms (Bruschke, 2020e).

**Risk and containment**

A second, longer lasting wave of infections was anticipated since SARS‐CoV‐2 arrival preceded the birthing season and juvenile mink kits could be expected to become gradually susceptible to COVID‐19 as maternal antibodies waned (Bruschke, 2020d). Rampant transmission among large numbers of susceptible animals coinciding with a period of increased human–mink exposure (birthing, weaning and scheduled core vaccination), plus the possibility of mutations, was deemed a persistent viral source which posed an increased risk to human health (Bruschke, 2020d). Therefore, given that surveillance and control measures had been insufficient to break the chain of transmission, the Dutch outbreak management team for zoonoses recommended stopping mink farming in the Netherlands after November's furring season (Bruschke, 2020c). Consequently, stamping out among infected units took place (69 farms up to December 2020), and the mink in remaining farms were pelted; effectively no farmed mink existed in the country by the end of 2020 (Bruschke, 2021). A legal ban on mink farming was brought forward on 8 January 2021 (Bruschke, 2021).

**Denmark**

**Initial detection**

The first positive farm was confirmed in June 2020 (Larsen, 2020a), at which point Denmark was reportedly the biggest mink producer in the world with an estimated herd of 17 million (WHO, 2020; WHO et al., 2021).

**Surveillance and spread**

Two further farms in the same area became positive, and it was decided to cull all animals in the infected farms and dispose of carcasses via rendering by the beginning of July. Among the three initially affected mink farms, two were epidemiologically linked to a local COVID‐19 positive nursing home (Larsen, 2020b). Virus sequencing indicated that people in the area with no contact to either farm or the nursing home were part of the same chain of infection (Larsen, 2020b). Consequently, a mandatory surveillance programme began by testing 10% of all farms in July (125 of 1140 farms (ECDC, 2020; Larsen, 2020c)). By early October, 94 premises in the northern and central Jutland region were confirmed infected (Larsen, 2020c). Control measures up to that point, including biosecurity and consecutive testing, had been insufficient; thus, all infected farms and those within a 7.8 km radius were culled affecting over 200 units (Larsen, 2020c). The virus had infected 207 farms up to 4 November 2020 (Larsen, 2020d).

**Risk and containment**

New unique mutations in SARS‐CoV‐2 sequences recovered from farmed mink and humans residing near to farms were discovered, and for a particular mink variant known as Cluster 5, antibody neutralization of the virus was reduced (ECDC, 2020; SSI, 2020; WHO, 2020). Such findings triggered a governmental mandate to cull the entire herd, including breeding stock. Because of the risk to public health, a de facto shutdown of the Danish mink industry is in place for 2021 although no general ban on future mink farming has been imposed (Larsen, 2020d).

**United States of America**

**Initial detection**

After respiratory signs and sudden death among mink were seen in the last week of July 2020 in two commercial farms located in Utah, infection with SARS‐CoV‐2 was confirmed (Davidson, 2020b, 2020c).

**Spread**

By October 2020, SARS‐CoV‐2 was present in 11 mink farms in the state of Utah with further spread to a farm in Michigan and another in Wisconsin (OIE, 2021b). By that time, at least 12 thousand farmed mink had died as a result of coronavirus infection (ECDC, 2020), of a national herd of 3.1 million (WHO et al., 2021). The following month, animals in a commercial unit in the state of Oregon presenting with inappetence, coughing and mild respiratory signs were confirmed SARS‐CoV‐2 positive, after recounts of COVID‐19‐positive in‐farm personal (Davidson, 2020a). Investigations carried out in a Michigan farm suggest mink‐to‐human transmission might have occurred (CDC, 2021). In contrast to the Netherlands and Denmark, a comprehensive surveillance system is yet to be implemented in the United States. A total of 16 farms have been confirmed infected up to the end of November 2020 (APHIS & USDA, 2021), and despite no subsequent reports to the World Organisation for Animal Health (OIE, 2021b), the outbreak is likely ongoing.

**Risk**

The response in the United States has been mostly limited to increased biosecurity, quarantine and disinfection of affected units. Culling has not been implemented, and testing is still restricted to symptomatic animals. PPE and testing of farm workers have recently been encouraged (CDC, 2021). One Health teams from the Centers for Disease Control and Prevention (CDC) are reported to be carrying out mink farm investigations (CDC, 2021). Interestingly, wildlife surveillance of areas surrounding infected farms in Utah, Wisconsin and Michigan, between August and October 2020, detected SARS‐CoV‐2 in a native wild animal for the very first time, among an unspecified number of wildlife species which tested negative (Miles, 2020). The single positive case was an asymptomatic free‐ranging American mink sampled in Utah, infected with a virus ‘indistinguishable from the virus characterized on the nearby affected commercial mink farm’ (Miles, 2020). Moreover, among 102 free‐roaming mammals captured outside affected Utah premises in August 2020, 11 presumed escaped mink presented high SARS‐CoV‐2 antibody titres, three of which also had high RT‐PCR cycle threshold detections (Shriner et al., 2021). Two further mink believed to have escaped from a farm in Oregon were reported as positive in December 2020 (Fine Maron, 2021). Such wildlife surveillance findings underline the risk posed by biosecurity breaches and insufficient control measures.

It has been reported that most Dutch mink farms developed a farm‐specific SARS‐CoV‐2 genomic signature which was employed to confirm animal‐to‐worker transmission events (Koopmans, 2021). Examination of the initial 16 Dutch farm outbreaks found evidence of SARS‐CoV‐2 infection in 68% of tested mink farm residents and employees (66 of 97 people tested), and nanopore sequences from infected mink and farm workers were phylogenetically grouped into five clusters (Oude Munnink et al., 2021). In contrast to the Dutch case, where spillover into the local community was not apparent, two of the three initially affected Danish farms were epidemiologically linked to a local COVID‐19‐positive nursing home, and moreover, viral sequencing indicated people in the area with no contact to either farm or the nursing home were part of the same chain of transmission (Larsen, 2020b). Aggregation of farms in the North Jutland region might explain such early spillover (BMJ & Dyer, 2020), as does the sheer numbers of mink involved: before the late November 2020 mink cull, there were roughly three times more mink than humans living in Denmark (approximately 17 million mink vs. 5.8  million humans). As recognized by the WHO (WHO, 2020), mink acted as a significant virus reservoir in Denmark and clearly contributed to ongoing transmission.

Spillover potential of mink is not limited to humans. The occurrence of mink escapes from commercial units is not uncommon and can lead to the introduction of SARS‐CoV‐2 into wild populations of mustelids and other animal species (Jo et al., 2020; Koopmans, 2021; Olival et al., 2020). In the United States, besides the presence of the virus in two mink presumed to have escaped from farms (Fine Maron, 2021; see Box 3) and seropositivity of a further 11 escaped mink (Shriner et al., 2021), the SARS‐CoV‐2 sequence recovered from a wild mink matched that found in an affected farm close to the sampling location (Miles, 2020; see Box 3). In January 2021, lymphatic tissue from two of 13 feral mink trapped in Valencia, Spain, showed a low RT‐PCR SARS‐CoV‐2 load later confirmed by Sanger sequencing (Aguiló‐Gisbert et al., 2021). These authors proposed such findings were not; however, the result of farm escapes and hypothesized two independent wastewater sporadic infection events among mink of stable riverside feral colonies in Spain had occurred (Aguiló‐Gisbert et al., 2021). Interestingly, SARS‐CoV‐2 infection from a yet‐to‐be‐clarified source was recently confirmed in four Asian small‐clawed otters (*Aonyx cinereous*) kept in an American aquarium (OIE, 2021b), an additional mustelid species with high susceptibility to natural infection (OIE, 2021a).

Co‐infection among susceptible mustelids with the betacoronavirus SARS‐CoV‐2 and species‐specific alphacoronaviruses (e.g. ferret enteric coronavirus (FRECV), ferret systemic coronavirus (FRSCV) or mink coronaviruses (MCoVs) which include the implicated cause of epizootic catarrhal gastroenteritis in mink; Stout et al., 2021) could, in theory, lead to further recombination and generation of novel recombinant viruses (Stout et al., 2021).

## 3 EVOLUTION OF VIRULENCE

Most concerning are the potential evolutionary consequences of allowing unchecked SARS‐CoV‐2 transmission in large, highly susceptible and densely populated mink populations. The adaptive trade‐off theory of pathogen evolution of virulence posits that there frequently exists a direct link between virulence—defined as pathogen‐induced damage to the host—and transmission, where virulence and transmissibility are positively associated with increasing within‐host replication (Acevedo et al., 2019).

The limits to virulence implied by the adaptive trade‐off theory both depend on a pathogen's mode of transmission and the epidemiological context in which transmission occurs. For instance, some pathogens are limited to an intermediate level of virulence because opportunities for transmission depend on a reasonably healthy and active host (e.g. HIV‐1 (Blanquart et al., 2016; Fraser et al., 2007)). For other pathogens, such as those transmitted by vectors (e.g. malaria (Mackinnon & Read, 2004)) or indirectly through the environment (e.g. cholera (Cressler et al., 2016; Ewald, 1991)), opportunities for transmission are less constrained by the health of the host and can thus evolve higher virulence. In the case of directly transmitted pathogens, evolutionary limits to virulence may be much more context dependent; if opportunities for transmission are abundant because of a constant supply of susceptible hosts in close contact, or where host populations are large and densely clustered together, evolution towards more severe virulence can go undeterred (Borovkov et al., 2013). For example, it has been proposed that the persistence of highly pathogenic avian influenza viruses in farmed populations is a consequence of the conditions created by large‐scale industrial poultry farming (Lebarbenchon et al., 2010).

Contexts which provide abundant opportunities for transmission therefore have potential to facilitate evolution of increased virulence. Indeed, the emergence of SARS‐CoV‐2 variants of concern (VOCs) from late 2020 (Fontanet et al., 2021) is associated with intense transmission (MacLean et al., 2021). Additionally, within‐host adaptation in chronic infection and responses to selective pressure to evade the immune system may have also played a role in the emergence of VOCs (MacLean et al., 2021). Notably, a recent study reported the occurrence of SARS‐CoV‐2 infection, recovery and three months after, reinfection among 75% of tested mink in a Danish farm, possibly as a result of continued viral replication within susceptible hosts with access to the premises (Rasmussen et al., 2021). Although surveillance of VOCs to date has been almost exclusively directed at humans, convergent evolution of potentially harmful mutations can occur in different host species and later spill back to humans.

The recent occurrence of emerging epidemics among farmed mink in several Chinese provinces (Fenollar et al., 2021), caused not only by new viruses—novel mink orthoreovirus outbreak in 2011 (MRV1HB_A) hypothesized to be the result of reassortment between human and swine reoviruses (Lian et al., 2013)—but also by viruses traditionally linked to other animal species—such as outbreaks of porcine pseudorabies virus (Aujeszky's disease agent) in 2014 (Wang et al., 2018); avian paramyxovirus type 1 (Newcastle disease agent) in 2014 (Zhao et al., 2017) and two highly pathogenic H5N1 avian influenza viruses (G15 and XB15) in 2015 (Jiang et al., 2017)—further highlights the susceptibility to infectious agents and zoonotic potential of American mink. Intensive farming practices, in conjunction with marginal nutrition and poor sanitation, enhance contagion among crowded, genetically homogenous mink (Fenollar et al., 2021).

Crucially, mink are an important species for generation of antigenically diverse respiratory viruses such as influenza (ECDC, 2020) and conceivably SARS‐CoV‐2. Recent phylogenetic analysis of the first 16 mink farms affected in the Netherlands hinted to a faster evolutionary rate of SARS‐CoV‐2 in the mink population in comparison with the evolutionary rate seen in humans (Oude Munnink et al., 2021). This might be explained by the comparatively higher metabolic rate of mustelids, multiple generations of infections before detection (Oude Munnink et al., 2021), plus the regular replenishment of naive animals and ease of transmission created by intensive production settings.

## 4 FUTURE DIRECTIONS: ENHANCED SURVEILLANCE AND ROBUST CONTROL

The current worldwide population of farmed mink is approximately 60.5 million (WHO et al., 2021; see Figure 1). Since the first COVID‐19 cases identified in mink in April 2020, over a year later, the number of mink farms reporting infection with SARS‐CoV‐2 by country is as follows: 69 in the Netherlands (OIE, 2021b); 290 in Denmark (Boklund et al., 2021; Fenollar et al., 2021); eight in Spain (OIE, 2021b); 17 in the USA (APHIS & USDA, 2021); 13 in Sweden (OIE, 2021b); one in Italy (OIE, 2021b); 23 in Greece (OIE, 2021b); one in France (OIE, 2021b); four in Lithuania (OIE, 2021b); three in Canada (OIE, 2021b); two in Poland (OIE, 2021b); and one in Latvia (OIE, 2021b).

Management of SARS‐CoV‐2 outbreaks has greatly differed between countries, ranging from a total ban on mink farming in the Netherlands, a temporary shutdown of the industry in Denmark, to quarantine, disinfection and increased biosecurity in the United States of America (see Box 3). In the Netherlands, the nationwide culls implemented in late 2020 and the bringing forward of an industry ban by 3 years (Bruschke, 2021) have decisively eliminated the risk associated with hosting a farmed mink reservoir for SARS‐CoV‐2. In Denmark, the 2021 industry shutdown was preceded by two epidemic phases that reached a peak in autumn 2020, closely following the COVID‐19 human epidemic curve over the same period (Boklund et al., 2021). Over the course of the epidemic in Denmark, different control strategies were implemented (Boklund et al., 2021; see Box 3), yet at least a quarter of infected farms, including a farm reinfection, were identified by regular tests of in‐farm personal and/or active surveillance within farms rather than by reliance on clinical signs or a rise in mortality among mink as prompts for testing (Boklund et al., 2021). In the USA, culls have not been implemented and case identification still relies on passive surveillance. Reports (see Box 3) of positive mink sampled in the field highlight the potential for establishment of this invasive species as a wild reservoir for SARS‐CoV‐2. Prospective comparative analysis of such varied SARS‐CoV‐2 mink outbreak responses would be most informative in the evaluation of its associated effectiveness in reducing viral spread.

Given the many consequences for public health of SARS‐CoV‐2 in farmed animals, a cohesive global response is needed consisting of surveillance of both spillover events and variants linked to mink farms, as well as effective control measures to combat unabated spread. A November 2020 statement by the World Organisation for Animal Health (OIE) urged countries to monitor and report COVID‐19 cases in animals (OIE, 2020). Early detection of infection should be prioritized on mink farms through both passive and active monitoring via regular testing of animals and staff, subsequent sequencing of positive samples, and prompt communication of results (Boklund et al., 2021). Moreover, multi‐species fur production units deserve special attention, particularly units raising mink, racoon dogs and/or rabbits. Inclusion of SARS‐CoV‐2 as an emergent multi‐species infection in the list of OIE notifiable diseases should be considered to promote case reporting.

In addition to vaccination of human populations and concomitant prioritization of mink farm staff, there are many reasons to propose parallel obligatory vaccination of farmed mink populations. Accordingly, the first COVID‐19 animal vaccine has been produced (Tétrault‐Farber & Vasilyeva, 2021) plus further mink vaccine candidates are under development. In late 2020, the Russian Federal Centre for Animal Health conducted clinical trials of a vaccine candidate (Vasilyeva, 2020). By March 2021, this immunological product was registered for use in carnivores—dogs, cats, foxes and mink (Tétrault‐Farber & Vasilyeva, 2021)—with initial pet vaccinations at veterinary clinics reported in May (BBC News, 2021). The Finnish Fur Breeders’ Association in conjunction with the University of Helsinki also announced work towards a vaccine for mink and raccoon dogs in January 2021 (FIFUR, 2021). Likewise, in January, the American drug company Zoetis supplied experimental vaccines under development for dogs, cats and mink, for emergency use in nonhuman primates at San Diego Zoo (Zoetis, 2021). Considering that mink farms harvest pelts destined to become luxury items of clothing, once these vaccine candidates reach commercial stage, industry profits could cover the costs associated with obligatory SARS‐CoV‐2 vaccination. In turn, vaccine profits raised by pharmaceuticals could be used to offset, for example, costs associated with expanding vaccine donation programmes to low‐ and middle‐income countries.

To complement vaccination, other integrated interventions should also be considered to minimize the risk of mink farms acting as biotic hubs for SARS‐CoV‐2 transmission and evolution. Structural improvements may include revisions of animal density, proximity of enclosures and airflow in commercial mink units. Existing biosecurity challenges should be overcome; for instance, the role of cats, bats, rodents and birds as potential carriers of SARS‐CoV‐2 between farms and into wildlife warrants investigation. Moreover, implementation of improved welfare standards would address concerns over the conditions imposed on this territorial, solitary species during intensive production (Xia et al., 2020), and at the same time, have the potential to limit viral spread.

In order to enhance surveillance and implement adequate measures to control this highly transmissible and adaptable multi‐host virus (MacLean et al., 2021), regulatory frameworks are needed. Imprecise estimates of the number, size and conditions of fur farms highlight the need for oversight, especially regarding the location of farms and surveillance activities (Koopmans, 2021). Surveillance of SARS‐CoV‐2 and other related viruses, not only of animal trade and markets but also of fur farms hosting COVID‐19 susceptible species, would further enhance efforts to trace SARS‐CoV‐2 origins and perhaps help prevent future pandemics (WHO Team, 2021).

## 5 CONCLUSIONS

The spread of SARS‐CoV‐2 in mustelid populations—particularly farmed mink—presents not just a zoonotic risk to humans, but more concerningly, a potential biotic hub where rampant transmission can facilitate the emergence of variants with enhanced virulence. The strategies to monitor and control the spread of SARS‐CoV‐2 among mink have varied widely, and the virus remains in circulation in mink populations throughout the globe. A more comprehensive and coordinated global response is required to tackle infection in mustelids, and consequently, mitigate the risk of future animal and human coronavirus outbreaks.

## CONFLICT OF INTEREST

The authors have no competing interests to declare.

## FUNDING INFORMATION

Biotechnology and Biological Sciences Research Council BB/M009513/1

## Data Availability

Data sharing is not applicable to this article as no new data were created or analysed in this study.
